# Social needs screening and referral in pediatric primary care clinics: a multiple case study

**DOI:** 10.1186/s12913-022-08692-x

**Published:** 2022-11-17

**Authors:** Rachel E. Massar, Carolyn A. Berry, Margaret M. Paul

**Affiliations:** Department of Population Health, NYU Grossman School of Medicine, 180 Madison Avenue 2nd Floor, 10016 New York, NY USA

**Keywords:** Social determinants of health, Social needs, Social risks, Screening, Pediatrics

## Abstract

**Background:**

Unmet social risks such as housing, food insecurity and safety concerns are associated with adverse health outcomes in adults and children. Experimentation with social needs screening in primary care is currently underway throughout the United States. Pediatric primary care practices are well-positioned to amplify the effects of social needs screening and referral programs because all members of the household have the potential to benefit from connection to needed social services; however, more research is needed to determine effective implementation strategies.

**Methods:**

To describe common implementation barriers and facilitators, we conducted 48 in-depth qualitative interviews with leadership, providers and staff between November 2018 and June 2019 as part of a multiple case study of social needs screening and referral programs based out of four pediatric ambulatory care clinics in New York City. Interviews were recorded, transcribed and coded using a protocol-driven, template-based rapid analysis approach designed for pragmatic health services research. In addition to analyzing content for our study, we delivered timely findings to each site individually in order to facilitate quality improvement changes in close-to-real time.

**Results:**

Effective implementation strategies included tailoring screening tools to meet the needs of families seen at the clinic and reflect the resources available in the community, hiring dedicated staff to manage the program, building strong and lasting partnerships with community-based organizations, establishing shared communication methods between partners, and utilizing technology for efficient tracking of screening data. Respondents were enthusiastic about the value of their programs and the impact on families, but remained concerned about long-term sustainability after the grant period.

**Conclusion:**

Implementation of social needs screening and referral interventions is dependent on contextual factors including the nature of family needs and the availability of intraorganizational and community resources to address those needs. Additional research is needed to prospectively test promising implementation strategies that were found to be effective across sites in this study. Sustainability of programs is challenging, and future research should also explore measurable outcomes and payment structures to support such interventions in pediatric settings, as well as aim to better understand caregiver perspectives to improve engagement.

## Background

The influence of social determinants of health (SDOH) on clinical outcomes and healthcare utilization is well documented [1–4]. Unmet social risks such as lack of or poor quality housing, food insecurity and safety concerns are associated with adverse healthcare outcomes and barriers to healthcare access [5]. The negative effects of unmet social risks are particularly consequential for children, with implications for development and lasting impacts to health and well-being [6, 7].

In the United States, primary care practices have become an increasingly common setting for programs aiming to mitigate the effects of social risks on health [8, 9]. Multiple professional medical organizations have come out in support of screening for social needs in clinical care settings, including the National Academies of Sciences, Engineering, and Medicine, the American Academy of Pediatrics, and the American Academy of Family Physicians [10–12]. Furthermore, according to the Kaiser Family Foundation’s Survey of Medicaid Officials, nearly half of state Medicaid agencies require social needs screening in Medicaid managed care contracts [13]. Experimentation with screening in primary care is currently underway across the country to systematically identify social needs [14], and there is an impetus to create strong linkages between primary care practices and community-based organizations (CBOs) to which patients can be referred to address these needs [15, 16]. Pediatric practices are especially eager to adopt these programs in order to promote healthy psycho-social development in children [17]. In addition, social needs screening in pediatric practices has the potential to impact family-level outcomes because all members of a household stand to benefit from connections to needed social services.

Despite the increased proliferation of social needs screening and referral programs throughout the US, there is much to learn about best practices for the implementation of such programs [18]. Previous studies assessing implementation of social needs screening and referral programs have found that integrating social needs screening into clinic workflows is both challenging and time-consuming [19], and coordinating referrals to external social services requires strong partnership and communication [20]. Establishing proven strategies to refine screening workflows and partnerships with external organizations will help to further the adoption of best practices in this rapidly expanding field. The primary objective of this study was to describe the common barriers and facilitators to implementing a social needs screening and referral program among pediatric primary care clinics.

Here we report our findings from a qualitative implementation study of four social needs screening and referral programs based in pediatric primary care clinics across New York City. Lessons learned from this research can inform the development and implementation of similar programs in pediatric clinics looking to establish sustainable and strong clinic-community partnerships.

### Program

The Partnerships for Early Childhood Development (PECD) collaborative was a grant program established by the United Hospital Fund in April 2017 to support the development and implementation of social needs screening and referral programs in pediatric primary care clinics across New York City. A core focus of the initiative was fostering partnerships between participating clinics and CBOs to which they could refer in order to address the social needs of children and their families. A total of 11 clinic-CBO teams initially participated in the first phase of PECD, which focused on program development and implementation beginning in April 2017. In June 2018, the second phase of the collaborative launched with 8 of the 11 clinic-CBO teams continuing their efforts to scale, sustain and evaluate their screening and referral programs. In November 2018, four clinic-CBO teams were selected to participate in the implementation study described in this paper.

### Study

Our research group partnered with PECD in order to evaluate the collaborative-wide implementation using a mixed methods approach. In the first phase of our study, we worked closely with the sites to develop an overarching logic model and core set of process measures to track key elements of implementation. In the second phase, once programs had been established for at least one year, we conducted a series of key informant interviews during site visits with four sites in order to assess key facilitators and barriers to program implementation, including factors associated with measuring program implementation, and potential for sustainability. In the third and final phase, we will assess programmatic impact with a parent survey (ongoing). We focus on findings from the qualitative interviews in this paper.

## Methods

### Sampling

For this multiple case study, we used purposeful sampling to select a subgroup of PECD sites based on qualitative assessments of two factors that we thought might influence program implementation: (1) intraorganizational resources in terms of funding and staffing (high/low) and (2) history of partnership between the clinic and CBO partners (established/new) with CBOs, in order to provide a view into implementation across varied sites. Each of the four “sites” was composed of a clinic and one or several CBO partners to which they routinely referred families with social needs. The clinics were housed in diverse settings, including a federally qualified health center, a large academic hospital, a small community hospital, and a large non-profit health system. We conducted a formative evaluation with explicit feedback loops. The four sites participated in two rounds of interviews during site visits that assessed adaptations at various stages of implementation. After each site visit, we provided site leaders with individualized memos describing their site’s specific implementation barriers and facilitators. The evaluation team also participated in monthly collaborative meetings with the funder as well as quarterly meetings with participating sites to present feedback on implementation barriers and facilitators across sites. During the second round of interviews, the evaluators noted changes in the programs that had taken place since the first site visit. We interviewed key informants from both the clinics and their CBO partners to gain an understanding of program operations from multiple perspectives. One site did not participate in second-round interviews due to loss of program leadership.

### Analysis

Informed consent from participants was not required because the activities do not meet the definition of research under 45 CFR 46.102(I) of the United States; however, all interview participants verbally agreed to participate voluntarily and gave permission for audio recording.

The evaluation team developed a semi-structured interview protocol with questions focused on program characteristics, inner and outer setting, including key facilitators and barriers to implementation, partnership between the clinic and CBO(s) to which they referred, and potential for sustainability beyond the grant-funded period. Two evaluators interviewed a total of 48 key informants across all four sites, including personnel directly involved with developing and implementing the program as well as other clinic and CBO staff. Interviews were conducted in-person between November 2018 and June 2019. With permission, interviews were audio-recorded and professionally transcribed. A deductive, protocol-driven rapid analysis approach [21] designed for pragmatic health services research was used to analyze qualitative data and deliver timely findings to each site individually in order to facilitate quality improvement changes. The research team developed a summary template outlining key domains that aligned with the interview protocol questions, to capture key themes related to implementation and illustrative quotes from the interviews. The summary templates were pilot tested among the research team to ensure there was agreement in the key domains captured. Two research team members then read through the interview transcripts and completed summaries using the template to document key themes and quotes within each domain. The summaries and corresponding quotes were transferred into an analytic matrix to compare and contrast the themes from each domain across the interviews from each site. The matrix was also used to compare findings within each site from the first round of interviews to the second round. The research team analyzed the summary data in the matrix and identified crosscutting themes within domains to yield widely applicable lessons relevant to implementation of similar social risk screening and referral programs.

## Results

In total, our evaluation team interviewed 48 individuals during two rounds of site visits (see Table 1). Interviews with the clinic-CBO teams focused on capturing the perspectives of individuals involved with the development and/or implementation of the program at each point in the workflow. While each clinic’s specific screening workflow and the team members involved varied, interviews with clinic teams generally included clinic leadership, providers, and front-line staff such as registration clerks and patient navigators who were responsible for identifying eligible patients and handing out or administering screening tools and/or making referrals to the CBOs. Interviews with CBO teams included those in leadership positions providing supervision of operations and front-line staff members responsible for receiving referrals and providing resources to clients. In some sites, front-line staff members were shared employees between the clinic and CBO partner.

**Table 1:** Key Informant Interview Participants (n = 48)

Site	Clinic leadership	Providers	Front-line staff	CBO leadership	Total participants
**Site 1**	3	2	6	1	12
**Site 2**	1	8	3	2	14
**Site 3**	2	4	4	2	12
**Site 4**	4	2	2	2	10
**Total**	10	16	15	7	48

Each of the four clinics was able to implement and sustain a social needs screening and referral program throughout the study period despite substantial differences in strength of community partnerships and availability of resources at the start of the grant period. The target population across all sites was caregivers of children with scheduled well-child exams, although target age range varied. Two sites targeted caregivers present at well-child visits ages 0 to 5 for social needs screening, while the other two expanded the age range with one screening at well-child visits up to age 13 and another screening at well-child visits up to age 18. Each team developed its own screening and referral workflows. Core elements of the programs were the same across sites, including the establishment of formal clinic-CBO partnerships, development of a screening tool and screening process within the clinics’ workflows, and establishing a referral process with CBOs. Sites differed in specific populations served, team structure, screening tool, common social needs among caregivers, and services offered by the partnering CBOs. Throughout the study period, and sometimes as a result of study findings, sites made adaptations to program structures and processes in order to improve implementation. The results presented here represent 7 overarching themes of barriers and facilitators that emerged across sites over the course of two rounds of interviews: (1) integration into clinic workflows; (2) screening tools; (3) staffing; (4) technology and tracking; (5) clinic-CBO partnerships; (6) caregiver engagement after screening; (7) and program value and sustainability (see Table 2).

**Table 2:** Common Barriers and Facilitators to Implementation of Social Needs Screening and Referral programs in Pediatric Primary Care settings

Barriers	Facilitators
**Caregiver Inability or Reluctance to Screening** Sites reported that a substantial proportion of caregivers were either unable to complete the screening tool due to low literacy or were reluctant to complete screening due to privacy concerns.	**Workflow Routinization** Sites achieved higher screening completion when they were able to integrate the screening process into the normal clinic workflow in a seamless manner. Strategies to pre-identify patients eligible for screening before their visit also proved effective.
**Lack of Technological Resources** Sites without resources to screen through electronic methods found paper-based screening to be cumbersome and data entry was time consuming. Providers were also not as aware of their patients’ social needs due to lack of automated EMR integration.	**Availability of Technological Resources** Sites that utilized tablet-based screening found it helpful for EMR integration and provider engagement in social needs discussions with families.
**Temporary/Part-Time Dedicated Staff** Sites that utilized part-time volunteers to assist caregivers in filling out screening tools found it difficult to handle frequent turnover and gaps in coverage.	**Full-Time Permanent Dedicated Staff** The consistent presence of a staff member dedicated to helping caregivers complete screening assessments was found to be extremely valuable.
**Lack of Shared Clinic-CBO Tracking System** Clinics and CBOs were generally unable to utilize the same data system due to HIPAA requirements, which made tracking and communicating about referrals challenging.	**Strong Clinic-CBO Communication** Establishing a system and cadence to facilitate communication between the clinic and CBO was seen as key to a successful partnership.
**Low Caregiver Engagement Post-Screening** Clinic-CBO teams often struggled with reaching caregivers after the screening process to engage them in referral and services.	**Shared CBO-Clinic Staff** Sites that employed a shared staff member solely dedicated to assisting caregivers referred by the clinic partner found that it was effective at minimizing caregivers who were lost-to-follow-up after being referred to services.
**Sustainable Funding** Sites did not have a formal plan in place for funding to sustain their programs after the grant period.	**Institutional Support** Sites that felt that their institution was open to ideas for finding a way to continue their program and potentially expand were hopeful they could sustain this work in the future.

### Integration into clinic workflows

One of the main challenges encountered by all clinics from the very beginning of program development was determining how to optimally integrate social needs screening into busy clinic workflows. Clinic staff described a period of time during which they piloted methods for screening administration, involving different staff members and strategies to routinize screening for the targeted population. One clinic began with front desk staff handing out the screening tool to eligible caregivers, but found that shifting the responsibility to medical assistants was more successful, given that they had more interaction with families and were able to devote more attention to helping them complete the screens.

*…in the beginning, the parents were given the questionnaires in the waiting area out front, and I think that that wasn’t going well because the front desk, they have a lot of responsibilities. There are a lot of people talking to them at the same time, and it’s just an extra thing for them to do that may have been lower on the priority list. So, now that it changed to the M.A. responsibility, it’s a lot better. -Nurse Practitioner*

This clinic also found that creating bound packets of all required screens for each age group was helpful for staff and boosted screening rates. Similarly, another clinic began “pre-screening” the appointment list the day before to flag which patients should receive a social needs screen when they arrived. Staff felt these small adjustments to the screening process made a huge difference for increasing screening rates while maintaining the regular workflows of the clinics.

### Screening tools

Each clinic site developed their own social needs screening tools, using previously validated tools as a starting point. After a pilot period, the sites modified their screening tools to include the most commonly identified needs of their patient population, as well as the needs for which community resources were available for referral. One site acknowledged that an issue arose when the screening tool in use included needs for which there were no services available for referral.

*So, we added things like do you need help with transportation? And I forget the other question that was added. But those two questions have already opened up problems because we don’t have answers for those questions. Like, everybody needs help with transportation. Well, what can I do about that? -Medical Director*

Clinic staff described common barriers to caregivers completing the social needs screening tools across sites. Low literacy level of caregivers was an issue mentioned by respondents in all four clinics as something that inhibited caregivers’ ability to answer questions accurately and independently. Another common challenge reported was that caregivers felt fearful and resisted completing the screening tool due to immigration status or privacy concerns. Some sites also reported noticing caregivers were experiencing “screening fatigue” from filling out numerous forms at multiple visits. One site noticed these screening challenges were contributing to “false negative” screens, where social risks were not identified at first, but were uncovered later through discussion with the provider or other staff.

*It does happen where they’re not comfortable writing in on a piece of paper or clicking it on the iPad. And so, if the providers sense that there’s something more, they’ll refer to me. And I’ll kind of look at the referral like, ‘There’s nothing here.’ And once the patient sits with me, they’ll just give all these other risk factors that we didn’t identify in the screener but were actually positive. -Clinical Social Worker*

The sites implemented screening differently based on resources available to them. One of the high-resource clinics utilized tablet-based screening supported by NowPow [22] software, while other clinics used paper-based screening only in the early stages of implementation, inhibiting their ability to use screening data in a timely manner. For example, during our first round of interviews, the lead provider at a low-resource site showed us a large stack of completed paper screens that had accumulated over the past week without staff available to assist with data entry.

### Staffing

Across sites, the availability of dedicated staff or volunteers to focus on the screening and referral program exclusively was viewed as key to successful implementation. Both high-resource clinics had a group of volunteer patient navigators who were readily available to assist families with screening and referral navigation. The volunteers were tasked with helping caregivers to understand the questions on the screening tool, explaining the purpose of the screen and allaying any concerns, including those related to immigration status. In comparison, during our first round of interviews, the two low-resource clinics were both seeking to hire someone in a full-time patient navigator role and struggled with implementation until they had secured the new staff. Clinic staff reported that having these dedicated volunteers or existing staff to assist caregivers with completing the screen was invaluable to program success. Volunteers or staff members who came from the community and spoke the same languages as the patient population were especially effective at building rapport and earning the trust of families.

*And, we’re in a Spanish-speaking community where it is hard—The language barrier is hard. So, having a couple of the [volunteer navigators] that do speak Spanish, they gave them that comfortability to open up. And they do come to them. Like, even when they’re finished with the iPad, sometimes they’re supposed to bring it to me, they’re like, ‘No. No, I want to talk to her. I haven’t seen her in a long time.’ --Front Desk Staff*

In addition to helping caregivers complete the screening tool, volunteers or staff members also assisted with navigation and referral to community resources. Once in place, dedicated staff members at the low-resource sites made direct connections to community resources and enhanced all aspects of program implementation, including implementation measurement. Prior to having dedicated staff members in place, the low-resource clinics had limited to no capacity to make referrals to community resources or follow-up with caregivers thereafter to confirm successful connection. At one clinic, prior to hiring a dedicated staff member, providers would give a paper handout to caregivers with the contact information for community resources; however, staff and providers had no knowledge of whether caregivers were actually connecting to resources. Having the capacity at the clinic to make the direct referral to resources for the caregiver and to follow-up with them afterwards was critical to implementation.

While additional staffing was necessary to facilitate screening completion and referral, the two high-resource clinics that utilized volunteers in their screening and referral workflows eventually reported challenges related to the temporary nature of the volunteers. These clinics experienced frequent turnover of volunteers, requiring recurrent recruitment and training. One clinic also reported a gap in coverage during evening hours that volunteers were not working. Finally, a respondent at one clinic mentioned concerns about volunteers having adequate training to handle sensitive mental health topics should they come up during screening or intake thereafter.

*So I think, to put it nicely, I think they just need a little bit more training on how to address a more serious situation, the proper wording to use, the proper way to address a family that may have some – I think of it from a different standpoint though because some needs obviously are such a wide range of needs. - Clinical Social Worker*

### Technology and tracking

The four participating clinics had different levels of technology resources available for screening. Three sites used paper screens, while one of the high-resource sites utilized tablets and a screening and referral software that integrated with their electronic medical record (EMR) system. This technology was seen as useful, making it possible for providers to see screening results automatically in their patients’ medical records. Conversely, providers at sites using paper screeners commented on the lack of feedback they received about screening and referral results. While the other clinics were able to manually enter screening data into the EMR systems, they expressed interest in being able to automate this process through technology. The clinic that utilized a screening and referral software found it to be helpful for generating lists of local resources for caregivers tailored to their home addresses.

### Clinic-CBO partnerships

An important aspect of the PECD project involved building strong clinic-community partnerships. Each clinic partnered with one or more CBOs to which they would refer caregivers for their identified social needs. Two of the sites had strong pre-existing histories of partnership with their CBOs, while the other two had only recently established their partnerships through this project. Interviews with clinic and CBO staff revealed common challenges and facilitators experienced by sites. Some differences between sites emerged that stemmed from whether partnerships were long-standing or new.

Respondents at all sites agreed that communication between clinics and CBOs was key to the program’s success. Each of the four teams had a plan in place and/or was developing a system as part of this pilot project to facilitate communication between the clinical and CBO partners to track the caregivers’ and/or patients’ post-referrals. The ability to share caregiver information openly between clinics and CBOs was made complicated, however, by HIPAA requirements. Each partnership established their own method of regular communication, such as using a shared Smartsheet spreadsheet, weekly in-person meetings, and email communications. While the two sites with long-standing partnerships benefited from a strong trust and commitment to working together, the two sites with new partnerships made up for lost time and by the time of our first round of interviews had already established strong methods of communication that were reportedly working quite well.

Three clinic-community partnerships utilized a shared staff member, employed by the CBO, who was solely dedicated to assisting caregivers referred by the clinic partner. This shared staff model was effective at bridging the gap between clinic and CBO and minimizing caregivers “falling through the cracks” after being referred to resources.

*And the idea was really to be able to bridge the referral from the hospital to [CBO], and also bridge the communication so that the client kind of doesn’t fall through the cracks in the referral process, and in some ways allows us to be able to provide more individualized follow-up with the family than we would have normally been able to do just in a general referral. So, we get referrals from lots of hospitals and lots of other nonprofits and community-based organizations. But this was really a way for us to develop a system of referral, and in some ways, kind of a VIP track for these referrals that were coming in from [clinic site]. -CBO Staff*

One CBO staff member discussed the importance of clinic staff having a full understanding and familiarity of what their CBO partner’s services were in order to make appropriate referrals to them. This particular CBO was narrowly focused on long-term home visiting programs for mothers. The CBO respondent noted that after providing clinic staff with more education on their organization, the caregivers referred to them were better informed and had needs they could address.

*And so, it’s been better since we had that like sit down and really been able to tell them what we do here. So, they’ve been able to send us the right referrals. So, the last like three months we’ve been getting referrals of people that are really wanting our course. -CBO staff*

One challenge across all clinic-CBO partnerships was the ability to “close the loop” on referrals, meaning CBOs communicating back to the clinic about the outcome of a specific referral. This challenge stemmed from the lack of a shared tracking and communication system and the many barriers associated with implementing one. One clinic began using a screening and referral software tool meant to streamline referrals and communication to CBOs, however, the CBO partner reported the software was not fully functional for sharing information freely.

### Caregiver Engagement after Screening

One of the most challenging aspects of the program identified by respondents, both on the clinic and CBO sides, was caregiver engagement. One interviewee explained that the neediest families are actually the hardest to reach and enroll in services due to the chaos they are experiencing in their lives.

*The problem is patient engagement…I always say we over-refer and under-go. As I was saying initially, our mentality is you have a problem, you go somewhere. Our patients, I—there’s something that’s lost in translation. We’re obsessed with referring. Our patients don’t think that way. So, these families will fall through the cracks. The neediest, you can’t get them because they’re just living chaotic lives. - Pediatrician*

A volunteer navigator at one site explained that completing intake in-person with caregivers after screening was much more successful than contacting them by phone after they had left the clinic, because they often were simply unable to reach them. Similarly, a CBO staff member noted that getting in contact with caregivers referred to them is a huge challenge.

*There’s been cases where we try to contact them multiple – we try to contact two – we say two times, but we call beyond and contact three or four times. And then if we can’t get ahold of them, then we’re like, okay, we tried to contact them, but unfortunately, we can’t, so we have to close the referral. - CBO Staff*

### Program value and sustainability

Respondents unanimously endorsed the value of addressing social needs for families, especially since their patient populations have high social risks. Several clinic staff members also spoke about how screening for social needs may strengthen family relationships with the clinic. They theorized that screening may make families feel that providers care about them and they may be more comfortable opening up about non-medical issues they are facing in their lives.

*Because sometimes, if you ask an open-ended question of a parent like, ‘do you have any issues that you want to talk about, or any questions?’, they may not feel comfortable bringing up certain issues, whereas filling out the screening tool, they seem to be more comfortable doing it in that way. And then we can react. So, I will be the first to admit that I rarely went into a very extensive discussion about issues of this kind. So, I think it’s made a dramatic difference, because I know a lot of parents would probably just not feel comfortable necessarily opening up about an issue to someone that perhaps they’re meeting for the first time and don’t really feel that they have a relationship with. -Pediatrician*

Furthermore, while some clinic staff reported the addition of the screening program was extra work for them, providers, including social workers and pediatricians, reported feeling that this program had taken some burden off of their work loads, and given them a deeper understanding of their patients.

When asked about the potential sustainability of the program beyond the grant funding, respondents agreed that they would like for the program to continue, but did not have a logistical plan in place that would replace the grant funding. Several clinic staff expressed hopefulness that their institutions would support the program following the grant period and were interested in exploring ways to align program activities with a value-based payment system. The two low-resource sites, in particular, were concerned that the full-time staff members they had hired would not be sustained after grant funding ended. One of the high-resource clinics was making plans to expand social needs screening to other clinics within their institution through additional external funding sources. Respondents at this site also stressed that institutional support would be necessary to make sustainability possible. CBO respondents underscored that funding for their organizations’ resources is also needed to sustain the capacity to serve direct referrals from clinics. At the time of the site visits, none of the CBOs reported having a sustainability plan in place for after the grant period.

## Discussion

This qualitative study sought to assess the common barriers and facilitators to implementing social needs screening and referral programs at pediatric primary care practices in New York City. Our findings show that adding a new screening process to busy clinics was challenging, but integration into the normal workflows and pre-visit identification of patients eligible for screening helped to improve screening completion rates. Technological resources for screening were valuable for efficiency and engagement of providers in the program through EMR integration. Additionally, caregiver reluctance to participate in screening was mitigated by the availability of dedicated program staff members, although part-time or temporary volunteers were found to be less beneficial than full-time permanent staff. Caregiver engagement after screening was challenging for all sites, though a shared clinic-CBO worker model helped some to minimize referrals falling through the cracks. Strong clinic-CBO communication was critical to successful partnerships, and teams had to overcome the lack of shared data systems. While no teams had a concrete plan for sustaining their program post-grant period, all expressed interest in continuing the program and some expressed hope their institutions would be willing to support the program long-term.

Social needs screening and referral programs in clinical settings are increasingly popular, yet successful implementation remains a challenge. Previous studies identified common challenges to integrating screening programs into busy clinic environments, including lack of perceived feasibility among providers [23] as well as insufficient time and dedicated resources to implement such a program [19]. Our findings reinforce and expand on these key lessons as respondents identified sufficient resources, including everything from dedicated staff, to capacity to integrate tools into the EMR, as the most essential component to implementation. Notably, although our sample included two relatively low-resource clinics, grant funding was available through the PECD initiative throughout the study period to support dedicated program staff. Consistent with the prior research, respondents highlighted the challenges to developing and maintaining effective partnerships between health care institutions and CBOs, two sectors which have historically operated separately from each other [20]. Despite two of the sites in our sample starting new partnerships with their CBO counterparts, the dedicated funding to CBO partners through the initiative helped to support program implementation across the two sectors. While these core components – availability of resources and capacity for effective multisector partnership – are critical for effective implementation, programs must also be responsive to highly localized contextual factors, which influence every facet of the program from the needs included on the screening tools, to workflows, and even prospects for sustainability beyond the grant-funded period. Findings across sites revealed several common implementation strategies corresponding to those described in the Expert Recommendations for Implementing Change (ERIC) project by Powell et al [24] to overcome barriers and support effective social needs screening and referral programs based in pediatric primary care settings:

***Change infrastructure.*** Sites piloted different strategies to integrate consistent social needs screening into clinic workflows without causing disruption to normal routines. Effective systems included identifying patients that needed to be screened and preparing screening packets in advance of visits, as well as identifying the right staff person(s) to engage caregivers in completing screens.

***Tailor strategies to fit the local population needs.*** While there are several validated screening tools available to assess social needs, we recommend tailoring these tools to reflect the languages and literacy level of the population, the most common needs of the population, as well as the community resources available via referral. Such a screen may need to be revisited periodically as needs and resources change in the local community.

***Create new clinical teams***. Volunteers were utilized by two high-resource sites to get the program up and running in the early phases of implementation, but high turnover among the volunteer pool and limited coverage led to gaps in availability. Across all sites, staff and providers agreed that having at least one paid, full-time staff person dedicated to implementation and monitoring of screening and referral processes was essential for a fully-functioning program.

***Build a coalition.*** In general, health care organizations and CBOs are not aligned ideologically or financially to collaborate with one another. While strong, cross sector partnerships are possible, establishing and maintaining such relationships is resource-intensive. For example, “closing the loop” on referrals made by clinics to CBOs is challenging given the lack of shared data systems. However, the relationships between the health care entity and its community partners are essential to effective implementation. In some sites, the dedicated staff overseeing the project were responsible for developing and maintaining relationships with community referral partners, while other sites used a shared worker model with community partners, both of which appear to be promising models. Clinic and CBO staff alike reported effective cross-agency communication as a facilitator to effective implementation.

***Develop resource sharing agreements.*** One of the main goals of screening and referral interventions is to “close the loop” with providers so that the health care team has an understanding of whether and to what extent their patients’ needs have been met. Clinic and CBO staff should establish a sustainable method of communication and ideally a shared data system to make sure caregivers are not falling through the cracks during the referral process.

***Change record systems and facilitate relay of clinical data to providers.*** Paper-based screening adds excessive work to screening and referral interventions, as all data must be manually entered into an electronic tracking document in order to be monitored over time. Integration with the EMR allows the health care team to readily retrieve and use data. EMR integration was a facilitator to both implementation and implementation tracking (i.e., screening rate, referral rate).

Due to the nature of qualitative methods, our findings are not representative of primary care settings at large. Our study was limited to New York City-based programs implemented in pediatric primary care settings and therefore may not be generalizable to other locations and patient populations; however, our findings are consistent with existing literature on implementation in other locales. We purposefully sampled four different clinics to maximize variability among key dimensions (intraorganizational resources and history of community partnership) to enhance generalizability. The clinic-CBO teams who participated in the PECD collaborative were early adopters in piloting innovative programs to identify and address social needs of their patients, and therefore highlighting their experiences is warranted.

## Conclusion

Despite substantial differences in intraorganizational resources and history of clinic-community partnership, each site was able to successfully develop and implement a social needs screening and referral program. Although each site’s program looked different on the ground, common implementation strategies worked for sites to overcome universal barriers to screening completion, tracking, and cross-organization communication. Future implementation research on social needs screening and referral programs in clinical settings should focus on prospectively rigorously testing the implementation strategies that were found to be common facilitators in this study.

While our study shows that implementation is feasible for different types of pediatric clinic-CBO teams, all sites including both clinics and CBO partners were concerned about sustainability in the long term, with many citing concerns over a lack of available payment mechanisms to support this work. Our findings indicate that these programs require real manpower to be effective, yet making a case for payment mechanisms to support these programs is challenging. Limited evidence suggests that cost-savings may be realized through social needs screening in pediatric clinical care settings [25]. However, in the case of our study, screenings were conducted during well-child exams and, thankfully, children are by and large a healthy population. Generally speaking, social needs interventions offered in a pediatric setting are intended to be “upstream” interventions and, therefore, we would not expect to observe meaningful cost savings in the short term. More research is needed to determine the link between such programs and measurable outcomes at the household or family level (e.g., parent stress) as well as to establish plausible payment reform mechanisms to support upstream social needs interventions. Finally, more caregiver-centered research is needed in the field to understand family perspectives on this work, and how screening and referral programs can be tailored to effectively engage them and impact family health.

## Data Availability

The datasets used and/or analyzed during the current study are available from the corresponding author on reasonable request.

## List of abbreviations

SDOH: Social determinants of health
PECD: Partnerships for Early Childhood Development
CBO: Community based organization
CFIR: Consolidated Framework for Implementation Research
EMR: Electronic medical record
